# High-accuracy virtual testing of air conditioner’s digital twin focusing on key material’s deformation and fracture behavior prediction

**DOI:** 10.1038/s41598-022-16511-w

**Published:** 2022-07-20

**Authors:** Shaohua Fu, Zhenping Wan, Weifeng Lu, Huaican Liu, Peng’e Zhang, Bo Yu, Jianming Tan, Feng Pan, Zhigang Liu

**Affiliations:** 1 Gree Electric Appliances, Inc., Zhuhai, China; 2 South China University of Technology, Guangzhou, China; 3 State Key Laboratory of Air-Conditioning Equipment and System Energy Conservation, Zhuhai, China; 4 Shanghai ShareFEA Engineering Technology Co., Ltd., Shanghai, China

**Keywords:** Mechanical engineering, Materials science

## Abstract

The concept of digital twin has been introduced for some time, yet one fundamental element of digital twin, digital material, has not been thoroughly studied. To interact with the physical product, the digital twin should always truthfully reflect the responses under various stimuli. In this paper, the deformation and fracture behavior of high impact polystyrene (HIPS) under the influencing factors of strain rate and stress triaxiality are studied to construct the material’s digital model. A digital twin of air conditioner product is further built and tested under virtual drop test. Comparing to experimental results, the acceleration curve, crazing induced whitening and the fracture events can all be captured by the digital twin. Our work demonstrates the importance of material characterization as an essential step to construct an accurate digital twin and shows a promising future of digital twin in virtual testing to replace traditional “trial and error” experiments.

## Introduction

Industrial manufacturing enterprises around the world are transforming into digital-driven enterprises, which requires a solid foundation of the digital twin (DT) in various aspects. During the product life cycle of a consumer product, its digital twin could be utilized in three phases. In the design phase, the digital twin could be tested to support the design of physical product. By conducting virtual tests of numerous types, the practicability of physical product^1^ could be evaluated; design factors like functions, aesthetics, budget, manufacture, reliability and recyclability, etc.^2–5^could also be considered. Nowadays, many manufacturing companies strategically build the DT of a new product to test and modify its DT virtually, which not only reduces the total cost but also increases the development speed of the physical product significantly. Successful examples include industrial sectors of automobiles^6,7^ aerospace^8,9^, manufacturing^10–13^ and robotics^14^. In the second stage of reconfiguration or redesign, the digital twin-based design validates system performance in a semi-physical simulation manner^1^. Similar to the rapid reconfiguration of commissioning manufacturing systems^15–18^, a product is also subject to reconfigure due to possible change of the structure, components, connection types and materials. Traditionally, the decision-making process is based on previous experience only, which results in the time-consuming “trial and error” loop of building physical prototypes, testing and reconfiguration until all the associated standards are passed^2^. Through optimization of the DT, the time for reconfiguration is greatly reduced and only a limited number of products need to be physically implemented serving the purpose to validate the accuracy of the DT and satisfy industry specifications^1,16^. The operation stage involves the interaction between the physical product and the DT through online parallel controlling, which enables the Internet of things (IOT) data integration, digital twin prediction, feedback and adjustment instructions to the physical product or large scale product–service systems^19,20^. It can be seen digital materials play a major role throughout the three phases; the benefits from the DT of any consumer product are highly dependent on the accurate characterization of the material behavior under various stimuli of mechanical, thermal, elector-magnetic fields and environmental influences, etc.^3,21,22^, which requires the attention of scientists and engineers to shift to the building block of the digital twin, the digital materials.

Among the widely available materials, rubber-modified polymer is quite unique as it satisfies multiple design objectives simultaneously due to its “all-around” properties. Ever since the discovery of rubber-modified polymers in late 1950s, this group of materials has been widely used in engineering applications. In particular, high impact polystyrene (HIPS) is widely used in home appliances, electronic devices and automobile parts^23–31^. Thus, HIPS is chosen as the model material to illustrate the importance of digital material for the accurate characterization of the digital twin. In domestic appliance industry, HIPS is often employed as outer casing acting as both high gloss appearance and structural support. After standard tests such as drop test, impact test and random vibration test, the product should not only retain its functions but also be free of whitening or visual cracks. This necessitates the building of the constitutive models of the involved materials such that a DT of the physical product could be constructed and tested virtually under various testing conditions. Due to its dual roles, the accuracy of the constitutive and failure model of HIPS becomes key to predict the mechanical behavior of the DT of domestic air conditioner at product development, production and operation stages.

Through recent decades, the benefits of rubber content on improving the mechanical performance of HIPS has been thoroughly studied^23–25^. A 5–10% weight ratio of rubber content could typically yield a 20–30% volume content of rubber through phase inversion during polymerization^24,25^ due to the formation of a special cellular or occluded structure, composed of many polystyrene inclusions embedded inside rubber^31^. Distinct from pure polystyrene, inside which concentrated crazes quickly connect to form critical crack and fracture, the rubber content in HIPS acts as both initiation sites and obstacles to craze; the latter prolongs the process for crazes to develop into critical crack length^32–34^. From microscopic view, crazes are commonly formed at equatorial zones of the rubber and propagate in direction perpendicular to tension. When the volume content of rubber exceeds about 15%, the close distance between rubber particles induces stress superposition, which allows thicker and longer craze to absorb more energy. The rubbers could further stop the craze from forming into long crack through crack tip blunting and force the crazes to form into distributed fashion enabling an enhancement of the ductility and impact resistance^25^. This phenomenon is widely observed under standard tensile tests of HIPS^35–38^. From macroscopic view, it has been shown that the threshold stress for crazing in HIPS is lower than the stress required for shear yielding^39^ in tension and the toughness under tension mode (mode I) is typically lower in comparison to that under shear mode (mode II)^40,41^. Thus, many researchers believe under most load-bearing scenarios the final fracture of HIPS should be induced by tension and accompanied with features of crazing^42,43^. However, there still lacks some evidence on how stress state other than tension would affect the fracture modes. Besides, the fracture pattern could also be altered based on different craze-crack interactions^24,25,44–47^. Higher loading rate would typically cause the craze opening rate to accelerate^48^ and altering the fracture pattern from features like parabola shapes at low crack extension speed to broken patch patterns at high crack extension speed; this phenomenon is found for both polystyrene^24^ and polypropylene^45^; whether similar phenomena could be found for HIPS remains unknown. Therefore, how factors like strain rate, stress state and fracture pattern could affect the deformation and fracture behavior of HIPS is still not fully explored and have become obstacles to construct detailed constitutive and failure model of HIPS, which are crucial for accurate virtual testing of the DT.

In this paper, the mechanical behavior of HIPS under the strain rate range of 0.001–100 s^−1^ and various designed stress triaxiality will be examined. Fractographic analysis of fracture surfaces of chosen stress state is conducted to further understand the damage-fracture mechanism of HIPS. A phenomenological constitutive and failure model of HIPS is constructed and incorporated in a digital twin of an air conditioner product with HIPS as outer casing and base plate. The product and its digital twin are further tested under drop test experimentally and virtually, respectively and compared to demonstrate the importance of digital materials in high accuracy prediction for digital twin.

## Methods

### Structure of the digital twin

**Figure 1:**
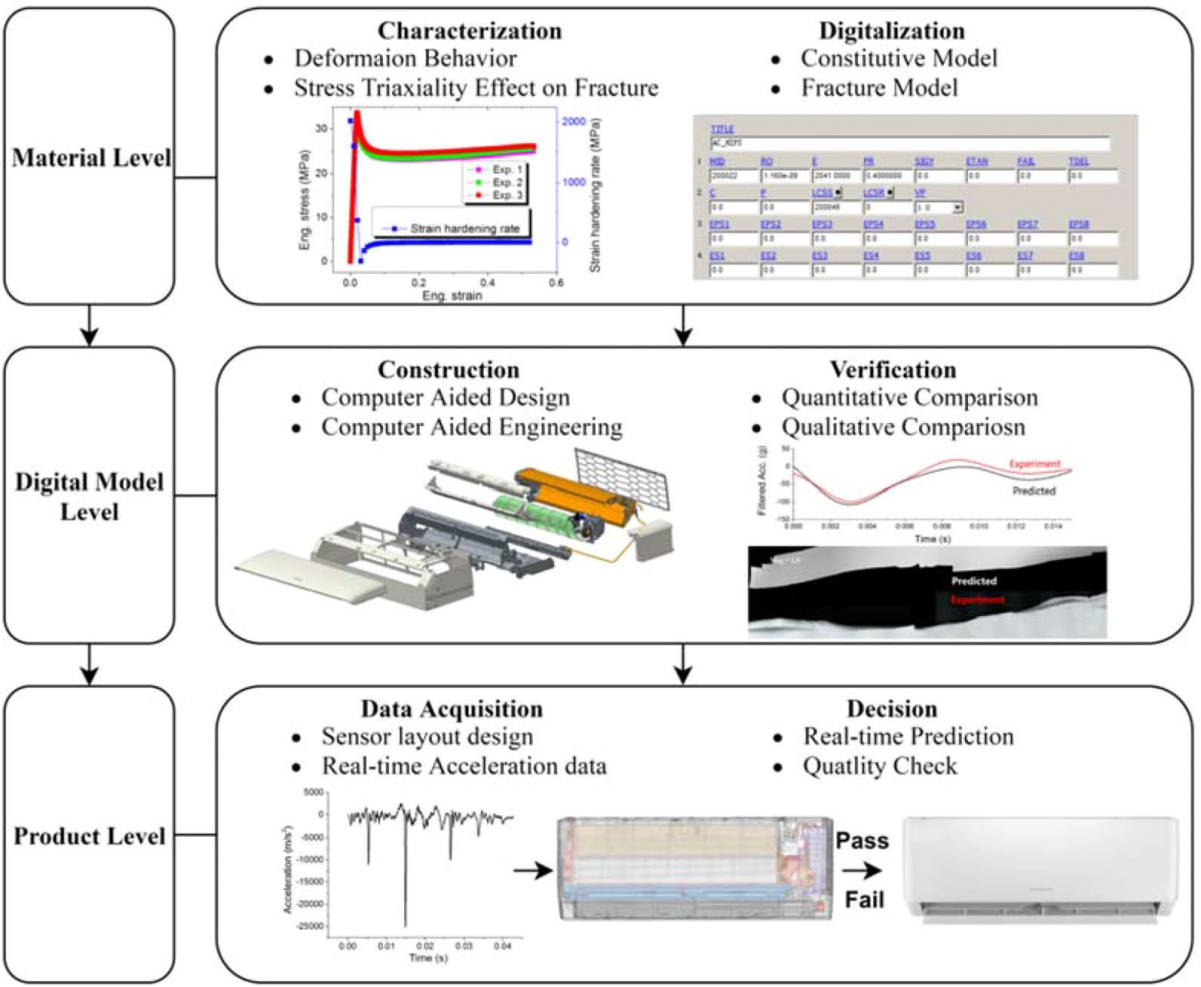
Schematic diagram showing structure of the digital twin of an air conditioner under mechanical stimuli.

In order to realize the possible deformation and fracture behavior of air conditioner product at various mechanical testing conditions, it is necessary to establish a digital twin system to truthfully reflect its responses. The main approach employed here is “bottom-up” as shown in Fig. 1: i.e. start from digital material to digital twin and physical product. At digital material level, the deformation and fracture behavior of HIPS is studied. Through characterization and analysis, the digital model of material could be constructed. At digital twin level, each part/component is dawn by computer aided design (CAD), converted, combined with material digital models and assembled into product through computer aided engineering (CAE). The digital twin is further verified under drop test in a consecutive manner. Quantitative comparisons are made based on experimentally collected data from physical product and data predicted by its DT. Qualitative comparisons are made based on crazing induced whitening, fracture path and fracture pattern observed after physical product test and the corresponding phenomena predicted by the DT directly or indirectly. At physical product level, real time data is captured by the product such that real time predication on the product deformation could be made to help identify the product quality and increase efficiency of the decision making process.

### Material and testing

**Figure 2:**
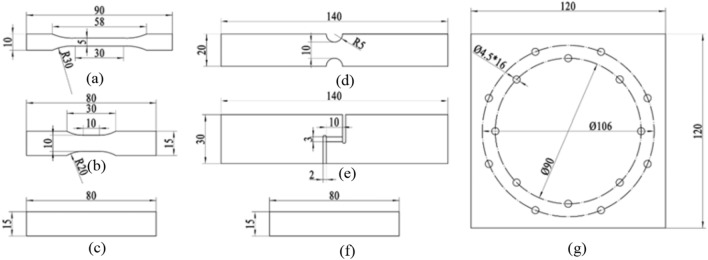
Schematic sketches of the specimens for: (**a**) static tension, (**b**) dynamic tension, (**c**) compression, (**d**) notched tensile, (**e**) grooved shear, (**f**) three-point bending and (**g**) puncture test (unit: mm).

Plastic sheets of HIPS are cut directly from the injection-molded outer casing of product and then machined through computer numerical control (CNC) technique to specific geometry of test specimen. Figure 2 presents the 7 specimen designs; for static and dynamic tensile test, the specimens are designed in accordance with ISO 527 and ISO 8256, respectively; for other tests the specimen designs are customized to include different stress states. The detailed types of experiments are listed in Table 1. The ideal stress triaxiality covers the range of − 0.33 to 0.66. The static tensile test is conducted using universal tensile machine (UTM) Instron 5982 with strain rate of 0.001 s^−1^. The dynamic tensile test is conducted using ZwickRoell HTM 5020 high-speed testing machines with strain rate ranging from 0.1 to 100 s^−1^. All the strain data is obtained through digital image correlation (DIC) technique. At least three repeated experiments of each type are conducted to ensure the accuracy of the experiments. The fracture path is observed by Keyence optical microscope (OM) and the fracture surface is observed using scanning electron microscope (SEM) Tescan VEGA3. The acceleration of the product during drop test is measured using Brüel & Kjær accelerometer sensors and analyzed using its dynamic signal analyzer.

**Table 1:** Summary of material experiments and associated stress triaxiality.

Specimen design	Test type	Stress triaxiality
(a)	Quasi-static tension	0.33
(b)	Dynamic tension	0.33
(c)	Compression	− 0.33
(d)	Notched tensile	0.44
(e)	Grooved shear	0
(f)	Three-point bending	N/A
(g)	Puncture	0.66

### Construction of digital twin

The physical product of air conditioner is subject to various mechanical stimuli including drop, impact and random vibration tests and other environmental conditions. It is not always practical to setup all the possible tests in a fast manner. By constructing the digital twin of air conditioner by assembling all the components, it allows the digital twin to be tested virtually under almost any scenarios. Product designers could further examine the performance of each component and turn results into efficient actions. Our method focuses on building the digital twin of air conditioner that can predict the mechanical responses of physical product under mechanical stimuli. The prototype DT is tested under drop test without cushion protection to mimic an extreme condition while being able to be tested under our testing facilities and collect data to be compared. In addition, two consecutive tests of the DT allow us to consider the effect of loading history on accumulative damage of the product, which typical simulation would not consider.

The digital material and DT are constructed and tested virtually under the LS-DYNA environment with explicit nonlinear finite element code. To construct the materials’ constitutive models, the hardening curve under different strain rate conditions are extracted from true-stress strain curve, fitted to proper formula with accuracy over 90%, extrapolated to 150% strain and incorporated in piecewise linear plasticity material model.

**Figure 3:**
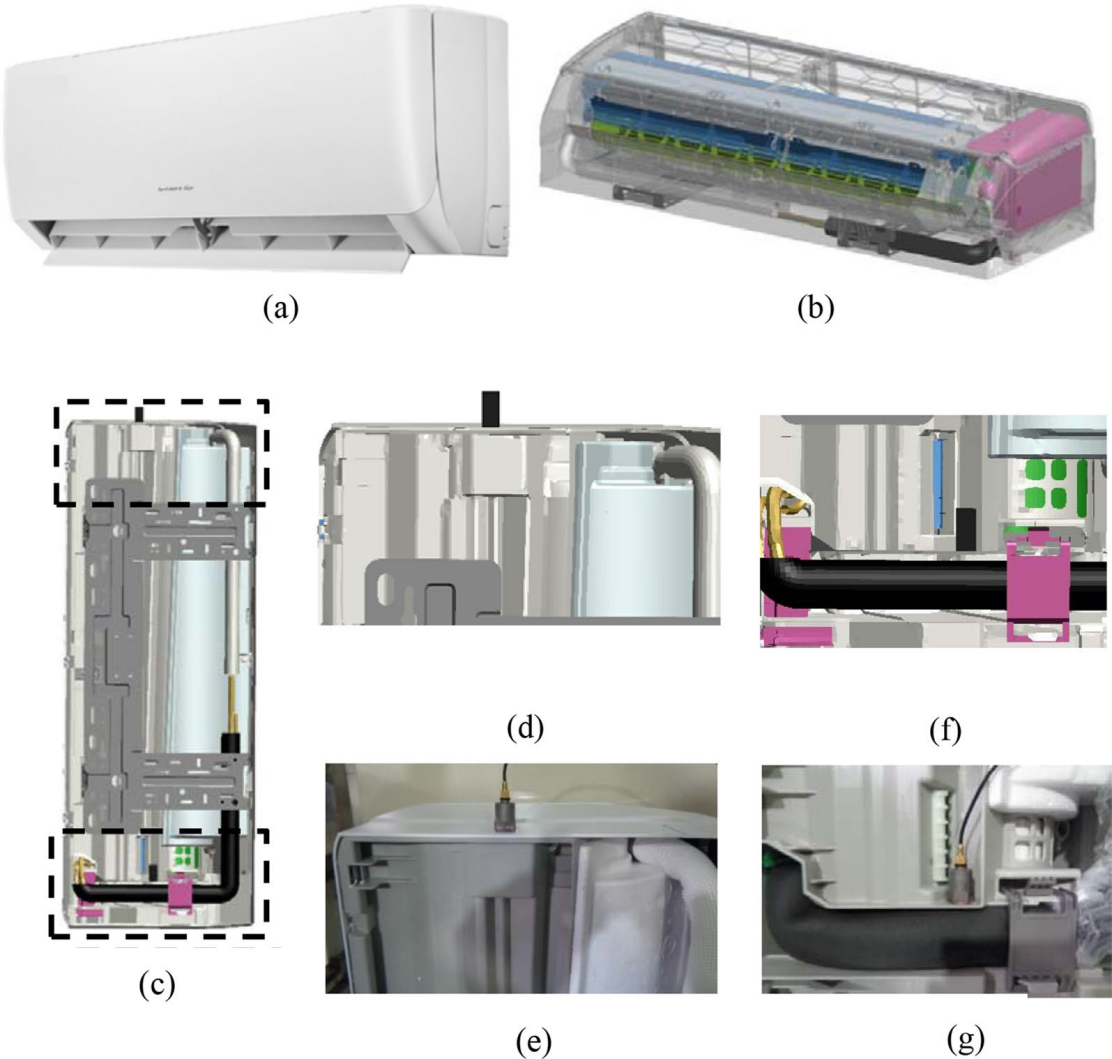
Images showing (**a**) air conditioner product, (**b**) frontal view of the digital twin, (**c**) rear view of the digital twin (accelerometer sensors are shown inside rectangular areas), position of top accelerometer sensor in (**d**) the digital twin and (**e**) physical product and position of bottom accelerometer sensor in (**f**) the digital twin and (**g**) physical product.

The failure model employed is generalized incremental stress-state dependent damage (GISSMO) model, which consider stress softening and accumulated damage to correlate failure events^49^. The key input is equivalent plastic strain to failure curve constructed based on the iterative algorithm of CrachFEM, a commercial software. The primary goal is to minimize the residual error between the calculated results of the finite element model and the force displacement curves from actual tests^50^. The digital twin of the air conditioner is constructed with all sub-systems and associated components as shown in Fig. 3a–c. The element size is optimally chosen as 3 × 3 mm after mesh convergence study. The overall model consists of 2 accelerometer element, 302,146 shell elements and 492,457 solid elements with a total number of 794,605 elements. Two consecutive drop tests at product level are designed. The first drop test corresponds to the vertical surface drop test, which aims to collect the acceleration data as the main parameter to verify the accuracy of the DT. Two accelerometer sensors are placed on the top and bottom of the physical product as shown in Fig. 3c–g. The second drop test is of edge drop type with a predefined inclination angle in order to create apparent crack and stress whitening such that the failure model can be verified. It should be noted the area of impact is meshed with smaller element i.e. 1 × 1 mm to better reflect the detailed crack path. A strain mapping technique is also employed to account for the plastic strain history from 1st surface drop test into 2nd edge drop test.

## Results

### Static and dynamic tensile test results

**Figure 4:**
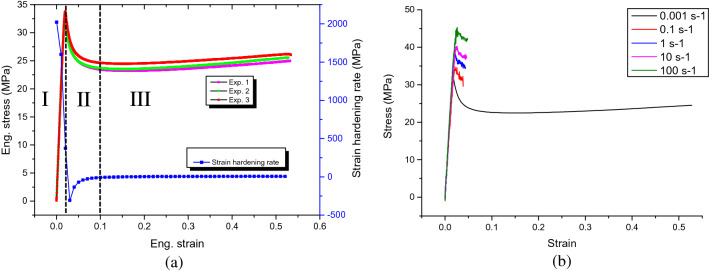
(**a**) Static stress–strain and strain hardening curves and (**b**) Stress–strain curves at strain rate range of 0.001 to 100 s^−1^.

The static and dynamic responses are fundamental to understand the mechanical behavior of HIPS. The stress strain curves of HIPS are shown in Fig. 4. The strain hardening rate curve is also extracted and analyzed. 3 deformation stages can be identified: stage I as the elastic region corresponding to strain level of 0–0.02, stage II as strain softening region with 30% reduction in stress corresponding to strain level of 0.02–0.1 and stage III as strain hardening region corresponding to strain level of 0.1 up to the strain at final fracture.

The stress strain curves of HIPS under strain rate ranging from 0.001 to 100 s^−1^ are shown in Fig. 4b. It can be observed as strain rate increases, the yield strength increases significantly whereas the ductility drops considerably due to the loss of stage III.

### Constitutive, failure model and validation

**Figure 5:**
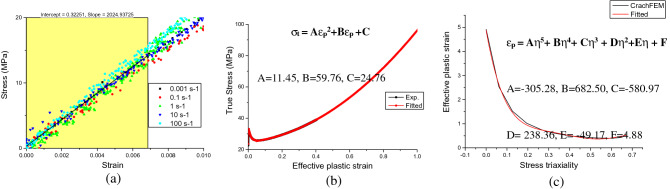
(**a**) Linear fit of elastic region (**b**) semi-experimental and semi-quadratic-fit of plastic region and (**c**) quantic polynomial fit of the plastic strain failure curve of HIPS.

In order to construct accurate constitutive and failure model, some key parameters required for FE analysis need to be identified. The elastic modulus is estimated by linear fit to the experimental data at all strain rates as shown in Fig. 5a and calculated as 2041 ± 113 MPa. It can be seen that the strain rate has almost no effect on the elastic modulus. It is found the hardening model can be constructed by semi-experimental and semi-fitting method^51^, i.e. the experimental data up to stage II is kept and a quadratic polynomial fit is added and extrapolated to 100% strain as shown in Fig. 5b. The plastic strain failure curve for input of GISSMO failure model is extracted from CrachFEM and fitted with quantic polynomial as shown in Fig. 5c.

**Figure 6:**
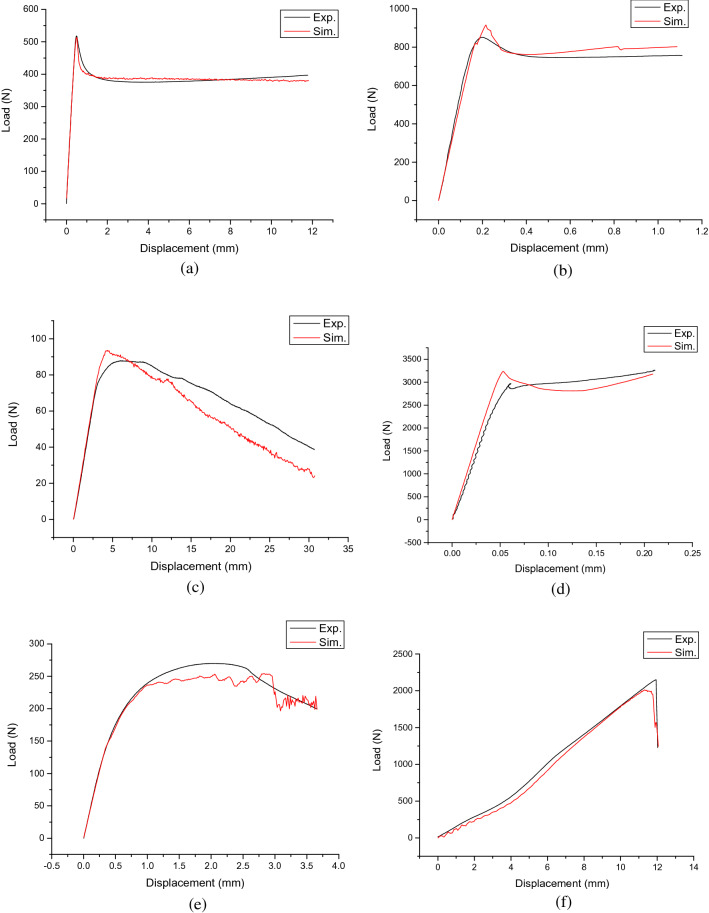
Experimental results in comparison to simulation results for (**a**) tensile test, (**b**) notched tensile test, (**c**) three-point bending test, (**d**) compression test, (**e**) grooved-shear test and (**f**) puncture test.

By putting the necessary parameters into the constitutive and failure model, a complete digital model of HIPS is constructed. To validate the model, the load–displacement curves obtained through 6 designed tests are compared to those calculated from simulations, respectively as shown in Fig. 6. It can be seen the results obtained under simulation are consistent with the experimental results at various tested stress state conditions.

### Fracture surface observations

**Figure 7:**
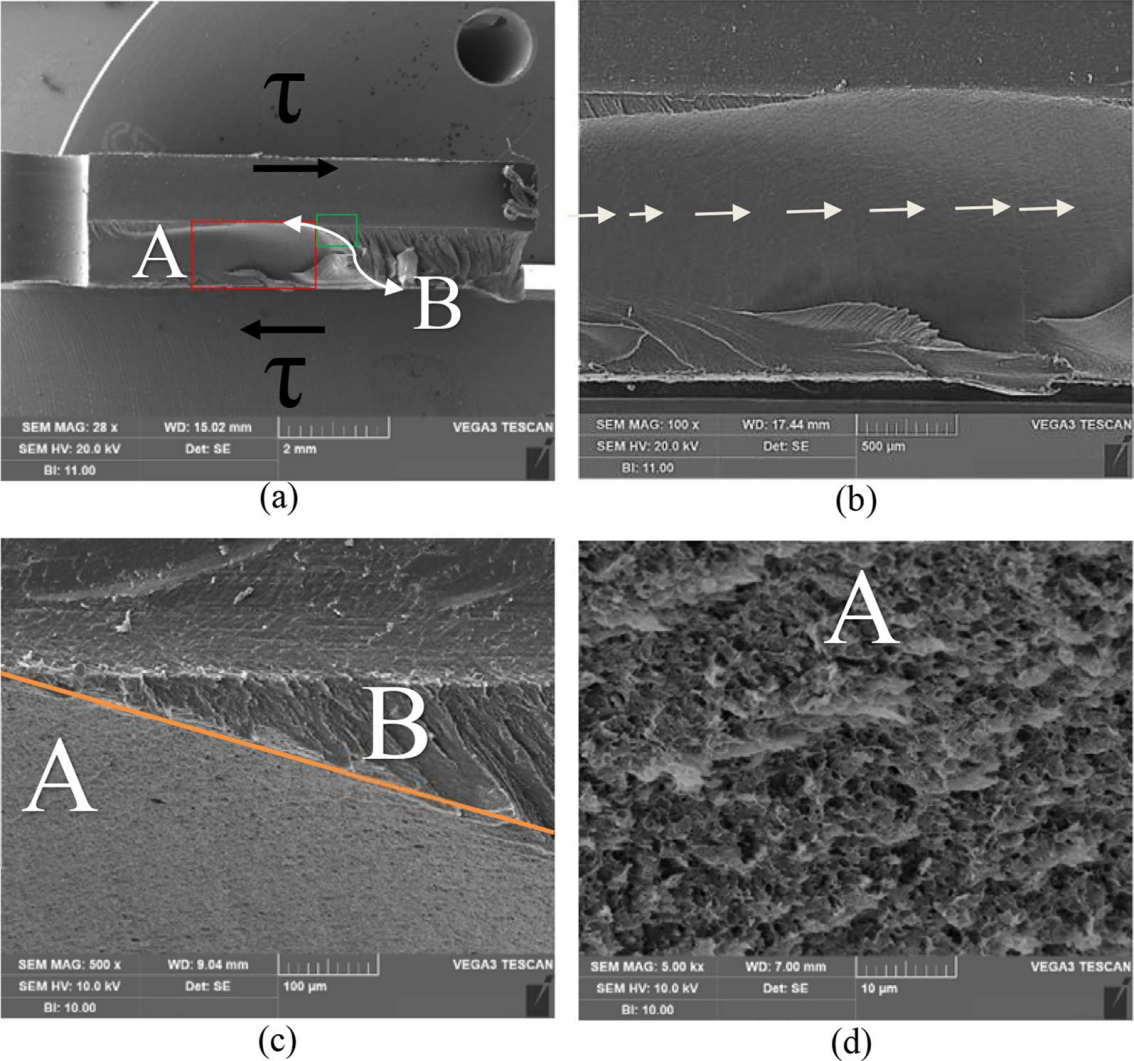
SEM images showing fracture surfaces of HIPS after shear test at (**a**) ×28, (**b**) enlarged view of red rectangle inside (**a**) at ×100, (**c**) enlarged view of green rectangle inside (**a**) at ×500 and (**d**) enlarged view of region A at ×5000.

To understand the damage-fracture mechanism of HIPS, the fracture surfaces from chosen tests are observed. The fracture surface of HIPS under shear test (Fig. 7) shows two completely distinct regions: region A has very fine features whereas region B has large fibrous sheets, which extend fully to the thickness of the specimen. Crack propagation lines are clearly visible as indicated by the white arrows shown inside region A in Fig. 7b. Inside region A, slanted torn features and numerous tiny voids are clearly seen during crack extension (Fig. 7c,d). Inside region B, the large fibrous sheets are resultant from the final instantaneous fracture (Fig. 7a,c), which was confirmed by the video recording.

**Figure 8:**
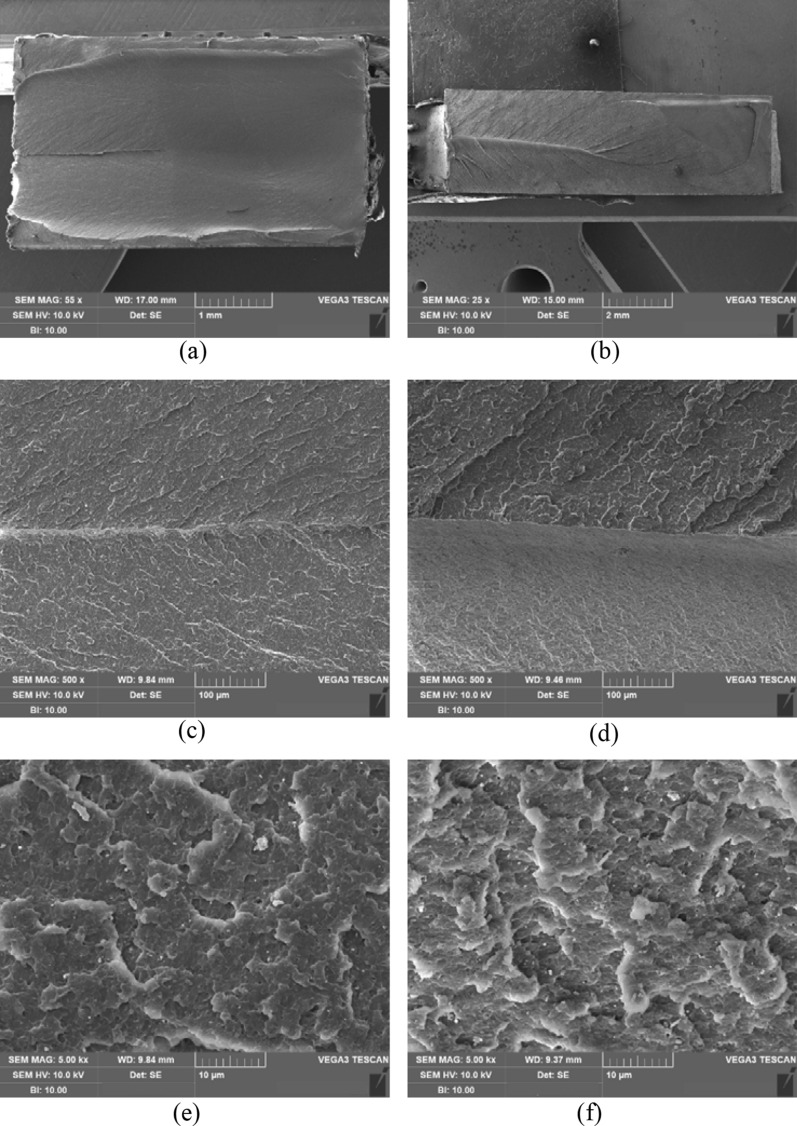
SEM images showing fracture surfaces of HIPS after tensile test at (**a**) ×55, (**c**) ×500, (**e**) ×5000 and after notched tensile test at (**b**) ×25, (**d**) ×500 and (**f**) ×5000.

Under both tensile test and notched tensile test, typical ductile fracture morphology is observed for HIPS. At low magnification, Fig. 8a,b indicates both samples illustrate crack interactions, whereas a ridge is formed when two crack front meet. At 500× magnification, it can be seen that the two regions besides the crack intersection line are very similar showing typical craze-crack interaction features (Fig. 8c,d). At 5000× magnification, a large number of fine incomplete half circle patterns are seen on the fracture surface for both cases. It is worth noting that the fracture surfaces under high stress triaxiality (Fig. 8) for tensile and notched tensile tests are quite different from that under low stress triaxiality for grooved shear test (Fig. 7).

### Virtual and experimental results of digital twin and physical product

**Figure 9:**
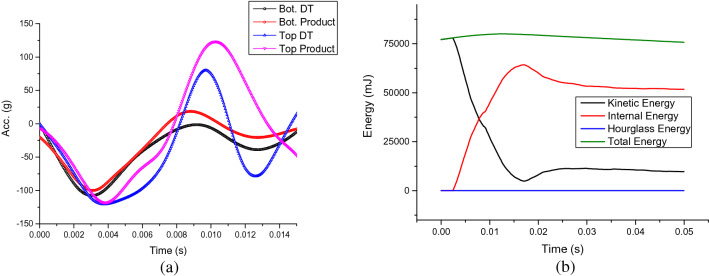
(**a**) Acceleration curves of digital twin and physical product of air conditioner unit (both filtered with SAE C60 standard) and (**b**) Energy map of the digital twin.

To further verify the digital model of HIPS, the DT of an air conditioner is constructed, tested virtually and compared with physical product’s experimental results. Figure 9a illustrates the acceleration curves obtained from both virtual and experimental surface drop tests. For the first peak of acceleration, the difference between the digital twin and the physical product for both top and bottom accelerometer sensors are quite small. Interestingly, the consistency of second peak for the bottom is better than the top, which might be due to the position of the sensor, where a stiffer component is placed on the bottom (Fig. 3g) than the top (Fig. 3e). The energy map of the digital twin, Fig. 9b indicates that overall, the total energy is conserved and the hourglass energy is close to 0. The kinetic energy from free fall drop is converted to internal energy of the system of the digital twin. From the perspective of acceleration prediction and energy conservation, the DT is verified.

**Figure 10:**
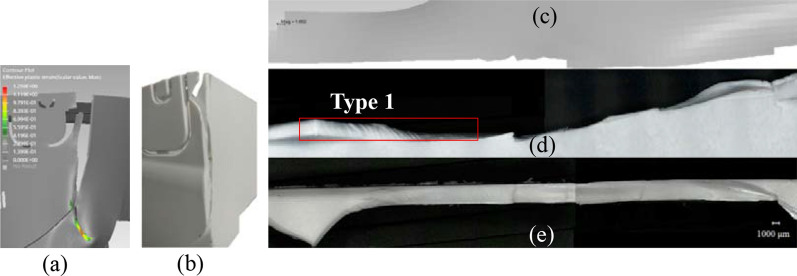
Fractured part (**a**) predicted by the digital twin, (**b**) photo-taken after drop test of physical product, main crack side-view profile (**c**) predicted by the digital twin, (**d**) photo-taken after drop test and (**e**) top view of crack under OM.

The second drop test is of edge drop type with a pre-defined inclination angle of 30° and height of 1 m between the impact edge and the ground. Apparent crack and stress whitening can be observed after test. Figure 10 illustrates the overall crack profile after actual edge drop test and that predicted by the DT. The major crack extension and crack initiation sites are well captured by the DT as shown in Fig. 10a,b. To better show the crack morphology, the part containing the major crack is cut and viewed under OM (Fig. 10d,e). Figure 10c shows the matching part containing this crack from the DT; it can be seen the detailed crack deviation points and path developments of the DT are all in agreement with those that appears in physical product (Fig. 10d,e). Furthermore, strands of fibrous sheets, which extend fully to the thickness is identified as type 1 fracture as shown in Fig. 10d.

**Figure 11:**
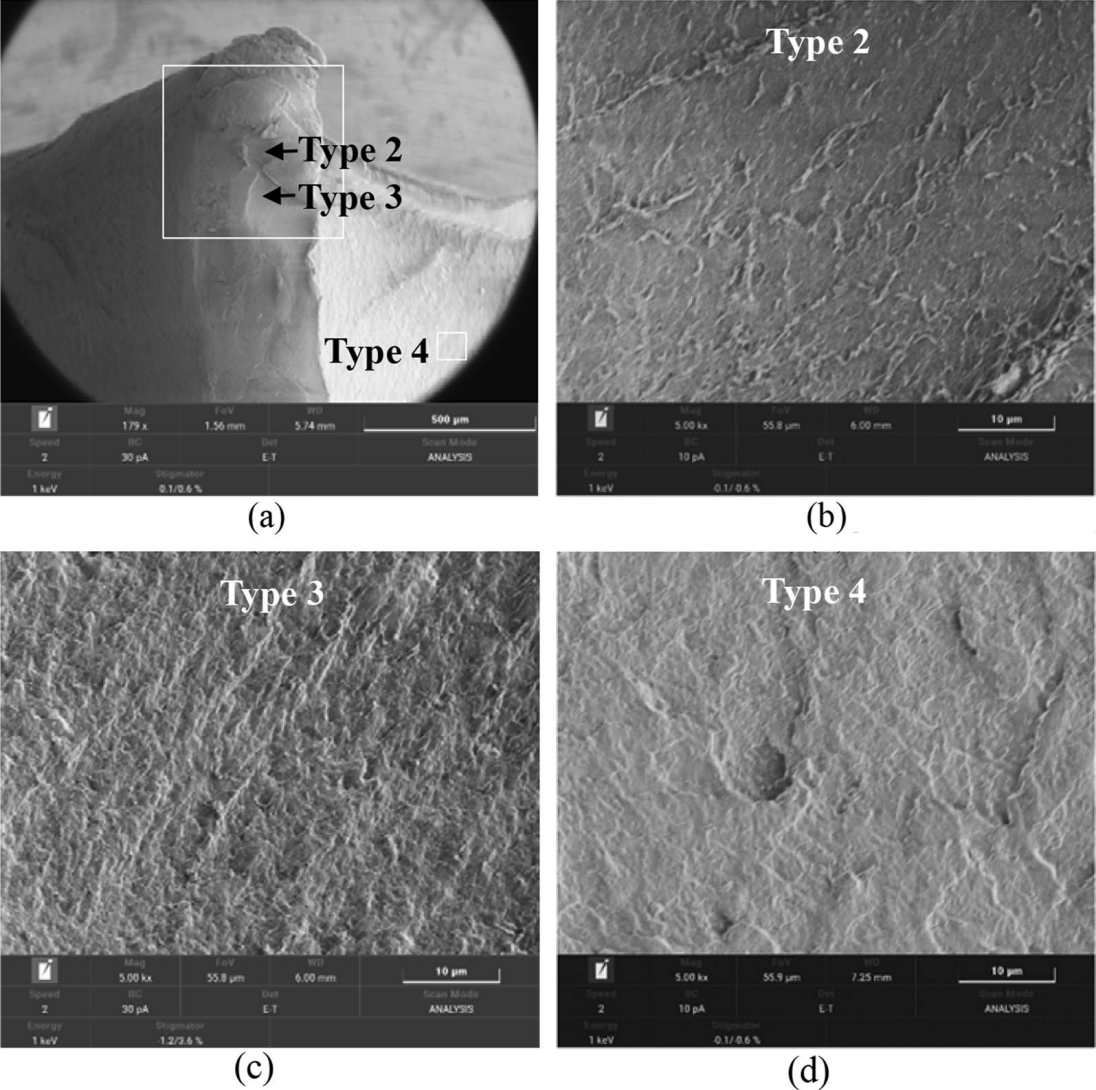
SEM images of crack origin showing (**a**) distinct regions of crack morphology are identified: type 2, type 3 and type 4 and close-up views of (**b**) type 2, (**c**) type 3 and (**d**) type 4 fracture patterns.

To further understand the fracture mechanism, the crack initiation site corresponding to the highest stress concentration is examined under SEM. Very different fracture features are observed as shown in Fig. 11. At higher magnification, it can be seen type 2 fracture surface is composed of typical mackerel or patch morphology (Fig. 11b), type 3 fracture surface contains highly plastically stretched fibrils with sharp tip and strong orientations (Fig. 11c) and type 4 fracture surface contains rugged surface with majority of half or unfinished circular steps (Fig. 11d).

## Discussions

### Deformation behavior of HIPS

**Figure 12:**
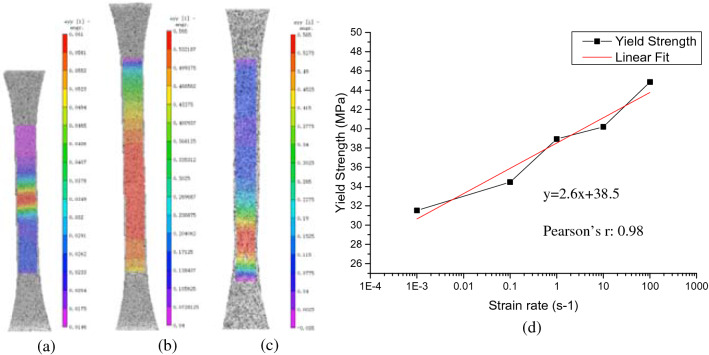
DIC strain contour of HIPS at (**a**) initiation of necking and (**b**) final stage of necking before fracture showing uniform necking; (**c**) DIC strain contour of ABS before fracture showing apparent necking/strain localization and (**d**) measured yield stress as a function of the logarithm of strain rate for HIPS at room temperature.

Under static tensile test, 3 deformation stages can be defined for HIPS, similar to polymers like PMMA, PC-ABS alloy^51^ and polycarbonate (PC)^52^. However, the mechanisms behind for those materials are quite different, this can be seen by the different necking developments and strain hardening rate curves. For HIPS and PMMA, crazing is the dominant deformation mechanism causing the initial necking to develop uniformly and total volume to increase before final fracture (Fig. 12a,b); since the strain extension is balanced by crazing only the strain hardening rate is quite low (Fig. 4a). For PC, ABS and PC-ABS alloy, the deformation is controlled by the competition between crazing and shear yielding such that apparent necking or strain localization (Fig. 12c) can be observed before fracture; shear yielding, which conserves the volume similar to strain hardening stage of metals, also causes the higher strain hardening rate, higher strength and lower ductility than those of HIPS^51^. This finding suggests that despite the price advantage of HIPS comparing to ABS, a reconfiguration prediction of the DT before material replacement is necessary due to their quite different mechanical properties.

With increasing strain rate, Fig. 4b shows the strength increases yet the ductility deceases with the disappearance of craze development stage, stage III. Since the resistance to polymer chain alignment causes strain hardening in the material at high strain rate^53,54^, this process can also be considered an energy-activated and rate-dependent process, an equation based on Eyring’s equation is expressed as^55^:

$${\sigma }_{y}=\frac{RT}{v}\mathrm{ln}\left[\frac{\dot{2e}}{\dot{{e}_{0}}}exp\frac{\Delta H}{kT}\right]$$

where σ_y_ = yield strength, R = ideal gas constant, T = temperature, v = activation volume, ė = strain rate, ė_0_ = constant pre-exponential factor, ΔH = height of potential energy barrier and k = Boltzmann’s constant.

This equation implies that there exists a linear relationship between σ_y_ and ln(ė). Such linear relationship is also found for the present HIPS (Fig. 12d), in agreement with findings on polymers like poly(viny1 chloride) PVC^56^.

It is speculated that the decrease in ductility at high strain rate might be related to 2 reasons: the increase in the density of micro voids or cavitation due to the less time for chain disentanglement^54^ and the possibility that rubber could not relax to build up enough stress to withstand the load with matrix together^55^. The increment of strength and altered hardening behavior were both taken into account in the material model, which helps in identifying crazing induced whitening as show in “Deformation induced visual whitening prediction”.

### Effect of stress triaxiality on fracture behavior of HIPS

The effective plastic strain to failure as a function of stress triaxiality (shown in Fig. 5c) is extracted by CrachFEM’s internal algorithm, which considers both normal fracture and shear fracture^50^. The curve is drawn through the iterative calculation results aiming to minimize the residual error between the calculated results of the FE model and the actual test force displacement curves, it can be seen the curve is very similar to the normal fracture curve indicating that at stress triaxiality greater than 0, thin sheet HIPS with plane stress states tends to fail by normal fracture mode, i.e. coalescence of micro-voids instead of shear band development for shear fracture. However, it seems the initial stress triaxiality and crack propagation speed could still influence the fracture pattern, our observation on the fracture surfaces of HIPS under different testing conditions brings some new insight on this issue.

**Figure 13:**
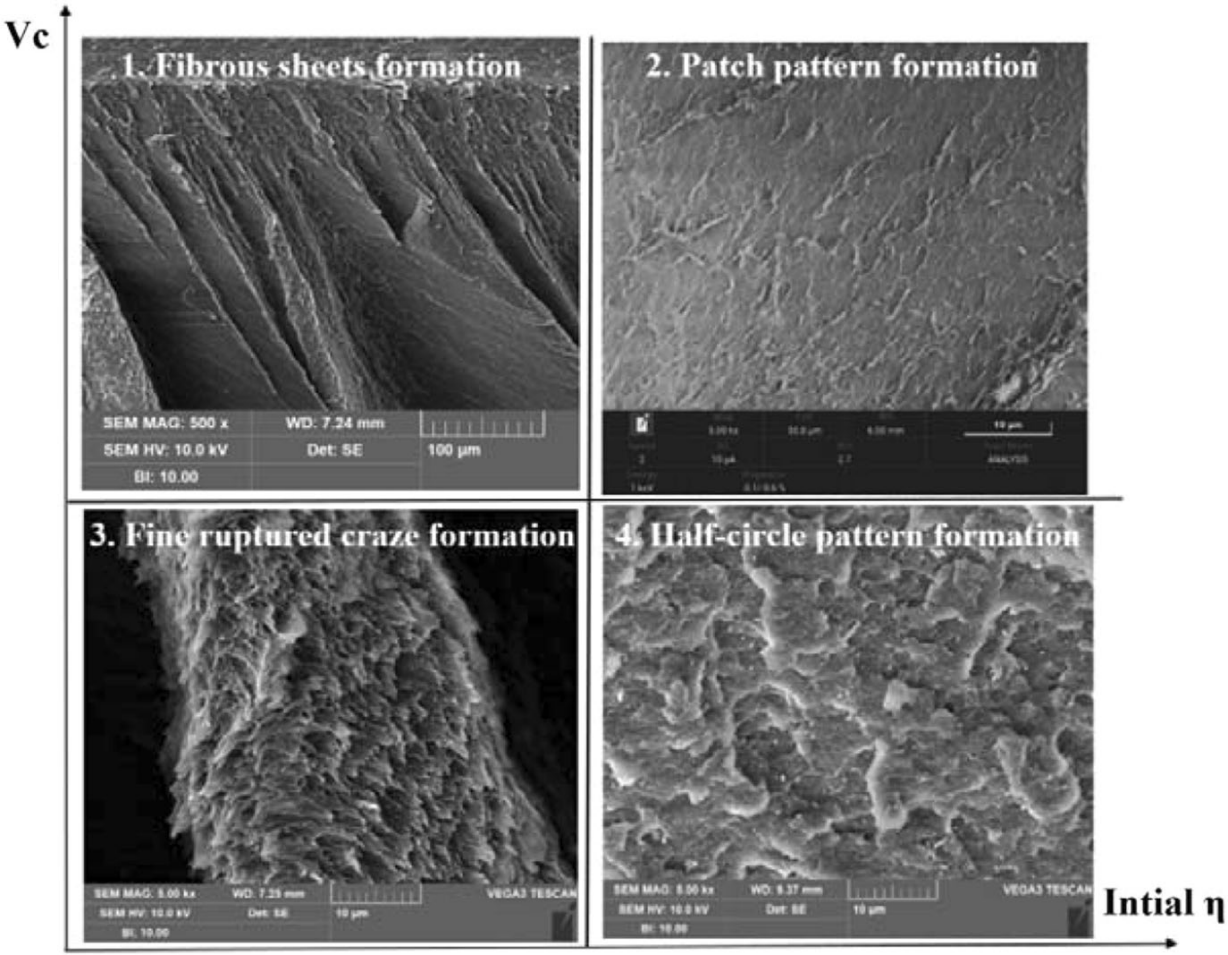
Proposed fracture pattern map of HIPS with abscissa as the initial stress triaxiality, η and ordinate as crack propagation speed, V_c_. (Note: type 1 is observed at ×500 and types 2–4 are all observed at same scale of ×5000 under SEM).

A fracture pattern map of HIPS resultant from the interaction of initial stress triaxiality, η and crack propagation speed, V_c_ is proposed and shown in Fig. 13. At quadrant of low η (shear mode) and high crack propagation speed, type 1 fracture surface contains large fibrous sheets (Fig. 7c, region B). It is speculated this morphology is due to the later tensile rupture of the shear band that formed during the beginning of shear test. Very similar phenomenon was observed in Ref.^57^, which subject atactic polystyrene to compression first and then tension. At quadrant of high η (0.33 and 0.44) and high crack propagation speed, type 2 fracture surface is very smooth showing patch patterns, which is consistent with findings on the fracture surfaces observed under high crack extension rate^39^ and low temperature^58^. At quadrant of low start η (shear mode) and low crack propagation speed (static condition), the fracture surface is of type 3, composed of very fine, highly stretched and fibrillated material (Fig. 7d) indicating that even at shear-dominate test the fracture is still controlled by craze-crack interaction at fine scale. It should be pointed out that this is due to the inability of actual test to maintain a pure shear condition and the stress triaxiality would change to be positive as test continues. Similar morphology can be seen in Ref.^25^. At quadrant of high η and low crack extension speed, the fracture surface is of type 4 showing half-circle pattern formations that are found after tensile and notched tensile test (Fig. 8), which might be resulted from interactions between growing circular crazing and a moving planar crack.

### Validation of air conditioner digital twin

The digital material model of HIPS is further utilized and validated in virtual drop test of the DT of an air conditioner product. The digital twin has been verified first by the correlation of the acceleration curves between predicted data and that measured by accelerometer and the energy conservation from surface drop test as shown in “Virtual and experimental results of digital twin and physical product”. The detailed deformation and fracture events predicted by the DT in comparison to those that occurred in physical product at second inclined drop test are shown in the following:

#### Deformation induced visual whitening prediction

For HIPS, it has been proved that crazing is the main deformation mechanism under most loading scenarios, thus the plastic strain should be an important indicator of the crazing induced whitening. Based on this understanding, three crazing criteria are established to predict the crazing induced whitening. 0.025, 0.05 and 0.1 plastic strain are chosen as the crazing criteria separately and the respective plastic strain contours of the DT are drawn for the two parts that experience major impact.

**Figure 14:**
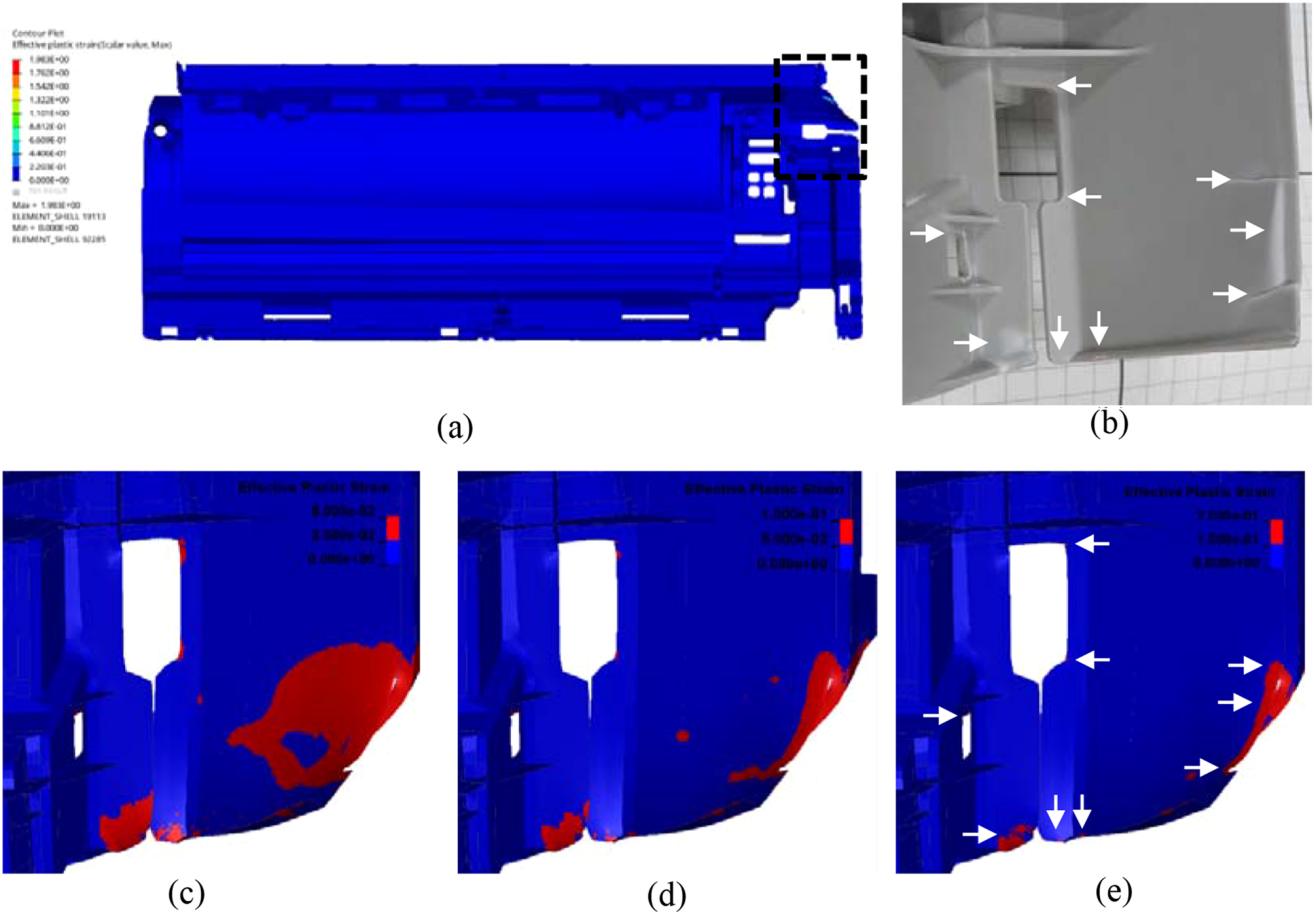
View of (**a**) base plate (closely observed area is shown inside rectangular), (**b**) whitening regions in real part in comparison to effective plastic strain FE map under crazing criteria of (**c**) 0.025, (**d**) 0.05 and (**e**) 0.1 (red regions: predicted whitening caused by crazing and white arrows: correctly predicted whitening regions).

**Figure 15:**
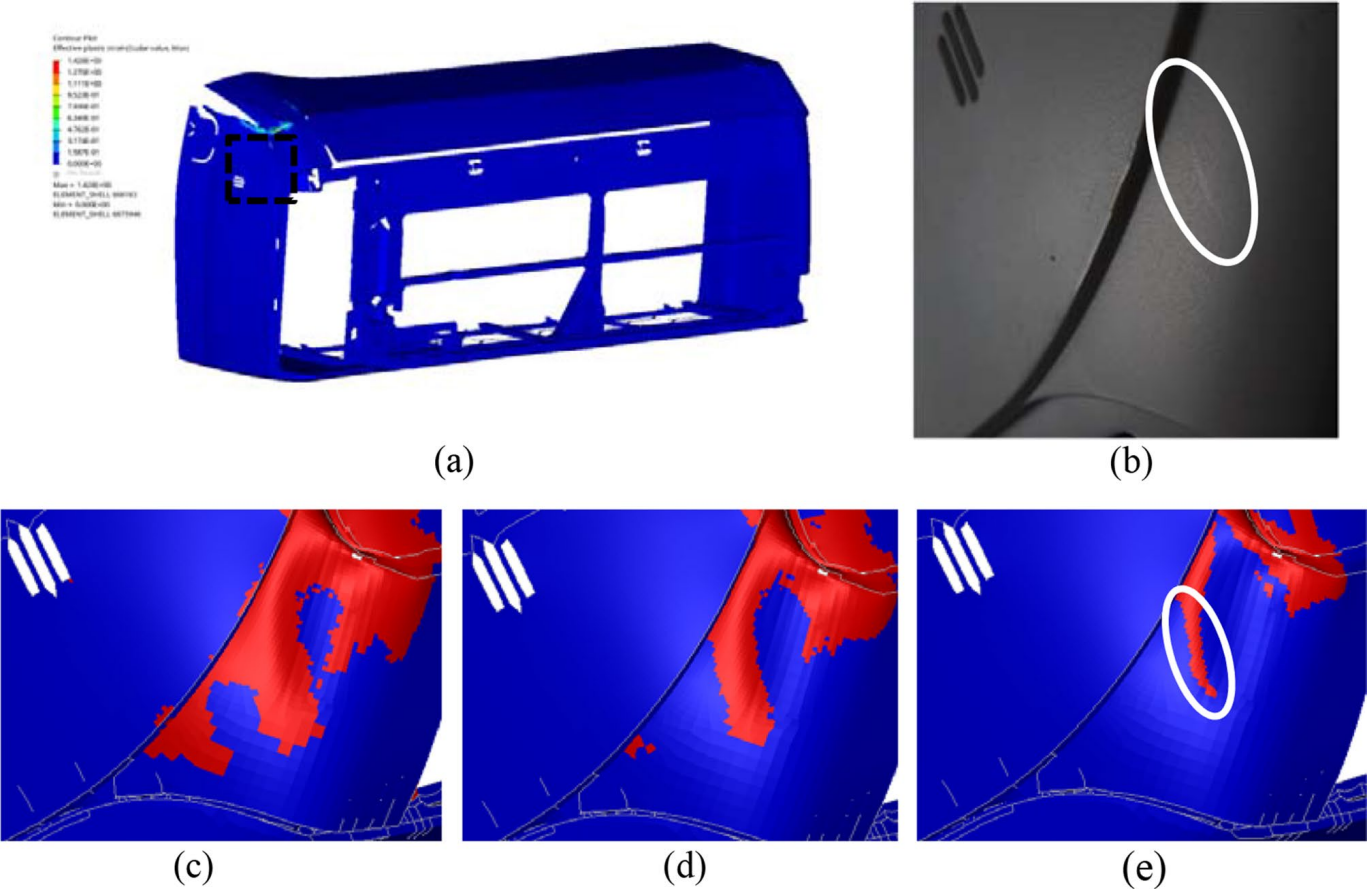
View of (**a**) outer panel (observed area is shown inside rectangular), (**b**) whitened region in real part in comparison to effective plastic strain FE map of outer panel under crazing criteria (**c**) 0.025, (**d**) 0.05 and (**e**) 0.1 (red region: predicted whitening caused by crazing, white circle: correctly predicted whitening region).

For the base plate, in comparison to the whitening area after the drop test (Fig. 14b), it is shown that under both criteria of 0.025 (Fig. 14c) and 0.05 (Fig. 14d) the predicted whitening areas (shown in red) are overestimated whereas only under the 0.1 criterion both the locations and sizes of the whitening zones are well captured, as indicated by the white arrows in Fig. 14b,e. Similar trend can also be seen for the observed area on the outer panel shown in Fig. 15. It is worth mentioning; the 0.1 plastic strain criterion is in accordance with the starting point of stage III (necking development) shown in Fig. 4a, correlating the accurate prediction of our DT to experimentally measurable material data.

#### Fracture prediction

**Figure 16:**
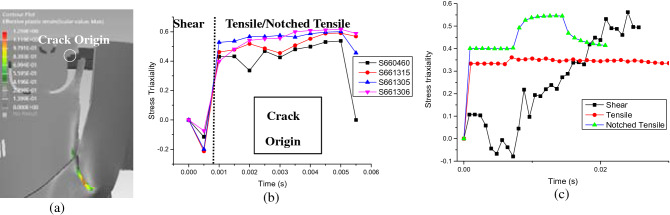
(**a**) Diagram showing the location of the eroded elements taken for crack origin, (**b**) corresponding stress triaxiality curves of the crack origin and (**c**) stress triaxiality curves of crack origins of individual tests of shear, tensile and notched tensile types.

As shown in “Virtual and experimental results of digital twin and physical product”, sound correlations can be established on the crack origin and crack extension profiles between the results predicted by the DT and that observed on the fractured part from physical product under OM. The upper fractured part (Fig. 10c) predicted by the DT matches the real fractured part (Fig. 10d,e) at majority of the crack-developing sites. In addition, the stress triaxiality values of those elements corresponding to the crack initiation site (Fig. 16a) are drawn in Fig. 16b. It can be seen the value of stress triaxiality changes in 2 stages: in the first stage it decreases to negative, increases to positive and then becomes stable at high stress triaxiality value in the second stage. Such varying stress state suggests that mixed modes of fracture might exist, which are confirmed by the different fracture patterns observed at crack initiation site under SEM (Fig. 11).

To further understand the 2-stage change of the stress triaxiality value (Fig. 16b) identified for crack initiation site during the drop test of our DT, the stress triaxiality curves for the first eroded elements under individual shear, tensile and notched tensile tests are all drawn in Fig. 16c for comparison. It can be seen that at crack initiation site the change of stress triaxiality in the first stage is in correlation to that of individual shear test i.e. the stress triaxiality value under both curves first decreases to negative value and then increases to positive value. This explains the type 3 fracture surface observed under both cases, which contains highly plastically stretched fibrils. In the second stage, the stress triaxiality curve becomes flat at high stress triaxiality value, which suggests a combination of tensile and notched tensile. Indeed, type 2 patch patterns and type 4 unfinished circular step patterns are observed at and near crack origin, respectively (Fig. 11) corresponding to the fracture surfaces observed at individual tensile and notched tensile test (Fig. 8). It is worth mentioning away from crack origin, Fig. 10 shows a portion of the fracture surface along the crack path is composed of large fibrous sheets, which corresponds to type 1 fracture pattern. According to^57^, the fibrous sheets inside atactic polystyrene are caused by tension of the shear band that formed previously under compression. This is also in agreement with our findings on the final fracture surface of HIPS after shear test (Fig. 7 region B), which resulted from the shifted stress state from pure shear to tensile and high crack extension rate.

Our work indicates that accurate digital material models could enhance the DT of consumer products at all 3 phases. At design phase, the feasibility of materials to be employed in components of consumer products could be evaluated in various aspects. Local deformation, fracture events of parts and cushioning property (acceleration) of product could all be captured as crucial guidance for adjustments of product design parameters. At reconfiguration stage, different product structure, materials and connection types could all be evaluated. Especially, due to the different deformation behaviors of HIPS and ABS, it is suggested a reconfiguration predication of the DT is necessary before material replacement. A physical product of the air conditioner is further implemented into drop test. High level of accuracy of the DT is achieved both quantitatively and qualitatively. In particular, the technique of microscopic characterization of the fracture surfaces is employed as an innovative verification method for testing the accuracy of the DT. At operation stage, based on the input of the physical product, the DT would predict and give instructions to the physical product. In our case, the accelerometer readings from physical product are treated as input to the DT, the DT could predict the material behavior of all parts and make judgments based on associated standards.

It should be pointed out that currently our research focus lies in the accurate prediction of the mechanical and fracture behavior of the DT only within the scenario of drop test, which limits a deeper interaction between our digital twin and product. In the next stage, the DT would be tested under other tests including inclined impact, random vibration and compression tests to verify and improve the accuracy of the DT. In addition, due to currently limited computing speed for simulation, the mechanical responses of the DT under various testing conditions of each test should be recorded and related to physically measurable mechanical stimulus such that a deformation database of the DT could be constructed and retrieved. For the physical product, wireless sensors would be installed on the product package to reflect the mechanical stimuli in “real-time” such that its the DT could reflect the deformation and damage concurrently to judge if the product passes the associated standards.

## Conclusion

In this paper, the deformation and fracture behavior of HIPS is investigated. The influences of strain rate and stress triaxiality are considered. The newly obtained knowledge is further incorporated in the material model and utilized to predict the detailed whitening and fracture phenomena of an air conditioner’s digital twin through virtual testing.

From material’s perspective, it is found the deformation behavior of HIPS under tension consists of three stages: elastic, strain softening and strain hardening. The uniform necking development up to final fracture observed under DIC suggests that crazing is the dominant mechanism, which differs from ABS with local necking before fracture. With increasing strain rate, the ductility is greatly reduced and yield strength increases linearly with natural logarithm of strain rate. In addition, although the fracture surfaces are resultant of crazing and crack front, the stress triaxiality and crack propagation speed are identified as two controlling factors for final fracture patterns. A fracture map is constructed based on present findings.

From product’s perspective, by incorporation materials’ digital models into the digital twin of an air conditioner, its deformation and fracture behavior under drop test can be predicted. For deformation prediction, a crazing criteria of 10% plastic strain, identical to the beginning of crazing development stage in tensile test, is identified and used to accurately predict both locations and degrees of the whitening for outer casing and base plate of the DT in agreement with those of physical product. For fracture prediction, a fracture model based on plastic failure strain and stress triaxiality curve is employed in the DT. The fracture path captured by the DT is also observed under OM at the same location of physical product. In addition, by extracting the stress triaxiality history of the fracture initiation site of the DT and relating to the fracture pattern map, mixed fracture patterns are predicted. This is verified by the fracture surface observation of the corresponding site on physical product under SEM, which consists of finely ruptured craze, patch pattern and half-circle pattern resultant from the change of stress state. Our work demonstrates the importance of material characterization for the high accuracy of the digital twin of consumer products and shows a promising future of the digital twin to replace traditional “trial and error” experiments, monitor “real-time” product life cycle and achieve predictive maintenance.

## Data Availability

The datasets generated during and/or analysed during the current study are not publicly available due to ongoing research but are available from the corresponding author on reasonable request.

## References

[1] Leng J, Wang D, Shen W, Li X, Liu Q, Chen X (2021). Digital twins-based smart manufacturing system design in industry 4.0: A review. J. Manuf. Syst..

[2] Lo CK, Chen CH, Zhong RY (2021). A review of digital twin in product design and development. Adv. Eng. Inf..

[3] Tao F, Sui F, Liu A, Qi Q, Zhang M, Song B, Guo Z, Lu SC-Y, Nee AYC (2018). Digital twin-driven product design framework. Int. J. Prod. Res..

[4] Sudarsan R, Fenves SJ, Sriram RD, Wang F (2005). A product information modeling framework for product lifecycle management. Comput. Aided Des..

[5] Ming XG, Yan JQ, Wang XH, Li SN, Lu WF, Peng QJ, Ma YS (2008). Collaborative process planning and manufacturing in product lifecycle management. Comput. Ind..

[6] Shcherba DI, Tarasov A, Borovkov A (2018). Developing of phenomenological damage model for automotive low-carbon structural steel for using in validation of Euroncap frontal impact. Mater. Phys. Mech..

[7] Li H, Li B, Liu G, Wen X, Wang H, Wang X, Zhang S, Zhai Z, Yang W (2022). A detection and configuration method for welding completeness in the automotive body-in-white panel based on digital twin. Sci. Rep..

[8] Tuegel E, Ingraffea A, Eason T, Spottswood S (2011). Reengineering aircraft structural life prediction using a digital twin. Int. J. Aerosp. Eng..

[9] Bayer V, Kunath S, Niemeier R, Horwege J (2018). Signal-based metamodels for predictive reliability analysis and virtual testing. Adv. Sci. Technol. Eng. Syst. J..

[10] Knapp GL, Mukherjee T, Zuback JS, Wei HL, Palmer TA, De A, DebRoy T (2017). Building blocks for a digital twin of additive manufacturing. Acta. Mater..

[11] Fedorko G, Molnar V, Vasil M, Salai R (2021). Proposal of digital twin for testing and measuring of transport belts for pipe conveyors within the concept Industry 4.0. Measurement.

[12] Jiang J, Li H, Mao Z, Liu F, Zhang J, Jiang Z, Li H (2022). digital twin auxiliary approach based on adaptive sparse attention network for diesel engine fault diagnosis. Sci. Rep..

[13] Yan D, Sha W, Wang D, Yang J, Zhang S (2022). Digital twin-driven variant design of a 3C electronic product assembly line. Sci. Rep..

[14] Jin T, Sun Z, Li L, Zhang Q, Zhu M, Zhang Z, Yuan G, Chen T, Tian Y, Hou X, Lee C (2020). Triboelectric nanogenerator sensors for soft robotics aiming at digital twin applications. Nat. Commun..

[15] Leng J, Zhou M, Xiao Y, Zhang H, Liu Q, Shen W, Su Q, Li L (2021). Digital twins-based remote semi-physical commissioning of flow-type smart manufacturing systems. J. Clean. Prod..

[16] Leng J, Liu Q, Ye S, Jing J, Wang Y, Zhang C, Zhang D, Chen X (2020). Digital twin-driven rapid reconfiguration of the automated manufacturing system via an open architecture model. Robot Comput. Integr. Manuf..

[17] Leng J, Zhang H, Yan D, Liu Q, Chen X, Zhang D (2019). Digital twin-driven manufacturing cyber-physical system for parallel controlling of smart workshop. J. Ambient. Intell. Humaniz. Comput..

[18] Zhao R, Yan D, Liu Q, Leng J, Wan J, Chen X, Zhang X (2019). Digital twin-driven cyber-physical system for autonomously controlling of micro punching system. IEEE Access.

[19] Leng J, Yan D, Liu Q, Zhang H, Zhao G, Wei L, Zhang D, Yu A, Chen X (2019). Digital twin-driven joint optimisation of packing and storage assignment in large-scale automated high-rise warehouse product-service system. Int. J. Comput. Integr. Manuf..

[20] Leng J, Yan D, Liu Q, Xu K, Zhao JL, Shi R, Wei L, Zhang D, Chen X (2020). ManuChain: Combining permissioned blockchain with a holistic optimization model as bi-level intelligence for smart manufacturing. IEEE Trans. Syst. Man Cybern. Syst..

[21] Tao F, Zhang H, Liu A, Nee AYC (2019). Digital twin in industry: State-of-the-art. IEEE Trans. Ind. Inf..

[22] Tao F, Qi Q (2019). Make more digital twins. Nature.

[23] Bucknall CB (1977). Toughned Plastics.

[24] Kinloch AJ, Young RJ (1995). Fracture Behaviour of Polymers.

[25] Michler GH, Balta-Calleja FJ (2012). Nano- and Micromechanics of Polymers Structure Modification and Improvement of Properties.

[26] Argon A, Cohen R, Mower T (1994). Mechanisms of toughening brittle polymers. Mater. Sci. Eng. A..

[27] Choi JH, Ahn KH, Kim SY (2000). Effects of the degree of graft on the tensile and dynamic behavior of high impact polystyrene. Polymer.

[28] Socrate S, Boyce MC, Lazzeri A (2001). A micromechanical model for multiple crazing in high impact polystyrene. Mech. Mater..

[29] Sahin T, Sinmazcelik T, Sahin S (2007). The effect of natural weathering on the mechanical, morphological and thermal properties of high impact polystyrene (HIPS). Mater. Des..

[30] Sharma R, Socrate S (2009). Micromechanics of uniaxial tensile deformation and failure in high impact polystyrene (HIPS). Polymer.

[31] Bucknall C, Cote F, Partridge I (1986). Rubber toughening of plastics. J. Mater. Sci..

[32] Bucknall C (2007). Quantitative approaches to particle cavitation, shear yielding, and crazing in rubber-toughened polymers. J. Polym. Sci. B..

[33] Maestrini C, Monti L, Kausch H (1996). Influence of particle-craze interactions on the sub-critical fracture of core-shell HIPS. Polymer.

[34] Tang C, Peng L, Li C, Shen W, Tsui C (2001). Experimental study on stable growth of crack and craze damage in HIPS under tension at room temperature. Polym. Test..

[35] Tang CY, Tsui CP, Shen W, Li CC, Peng LH (2001). Modelling of non-linear stress–strain behaviour of HIPS with craze damage in tensile loading–unloading process. Polym. Test..

[36] Castellani CML (1990). Rubber-like tensile behaviour of yielded high-impact polystyrene. Polymer.

[37] Tang CY, Tai WH, Lee WB (1996). Modeling of damage behaviors of high impact polystyrene. Eng. Fract. Mech..

[38] Lee C, Lu M, Chang F (1993). Fracture toughness of high-impact polystyrene based on three j-integral methods. J. Appl. Polym Sci..

[39] Lauterwasser BD, Kramer EJ (1979). Microscopic mechanisms and mechanics of craze growth and fracture. Philos. Mag. A..

[40] Williams JG (1984). Fracture Mechanics of Polymers.

[41] Kwon H, Jar P (2005). Fracture toughness of polymers in shear mode. Polymer.

[42] Husaini, Kishimoto K, Notomi M, Shibuya T (2001). Fracture behaviour of PC/ABS resin under mixed-mode loading. Fatigue Fract. Eng. Mater. Struct..

[43] Li J, Zhang X, Recho N (2004). J-Mp based criteria for bifurcation assessment of a crack in elastic–plastic materials under mixed mode I–II loading. Eng. Fract. Mech..

[44] Bucknall CB, Smith RR (1965). Stress-whitening in high-impact polystyrenes. Polymer.

[45] Luo TYW (2003). Computer simulation of conic-shaped patterns on fracture surfaces of polymers. J. App. Polym Sci..

[46] Yilmaz T, Sahin T, Sinmazcelik T (2009). Fracture characteristics of high impact polystyrene under impact fatigue loadings. J. Mater. Sci..

[47] Heyden S, Conti S, Ortiz M (2015). A nonlocal model of fracture by crazing in polymers. Mech. Mater..

[48] Tijssens M, Giessen E, Sluys L (1999). Modeling of crazing using a cohesive surface methodology. Mech. Mater..

[49] LS-DYNA Keyword User's Manual Vol. II Material Models, Livermore Software Technology (LST) (An ANSYS Company, 2020).

[50] MF GenYld+CrachFEM 4.2 User's Manual, MATFEM Partnerschaft Dr. Gese & Oberhofer (2014).

[51] Helbig, A. H. M. Modeling of Crazing in Rubber-toughened Polymers with LS-DYNA®, 15th Int. LS-DYNA User Conf. (2018) https://www.dynalook.com/conferences/15th-international-ls-dyna-conference.

[52] Fleck NA, Stronge WJ, Liu JH (1990). High strain-rate shear response of Polycarbonate and Polymethyl Methacrylate. Proc. R. Soc..

[53] Arruda EM, Boyce MC, Jayachandran R (1995). Effects of strain rate, temperature and thermomechanical coupling on the finite strain deformation of glassy polymers. Mech. Mater..

[54] Siviour CR, Jordan JL (2016). High strain rate mechanics of polymers: A review. J. Dyn. Behav. Mater..

[55] Ward SIM (2013). Mechanical Properties of Solid Polymers.

[56] Bauwens JC, Bauwens-Crowet C, Homes G (1969). Tensile yield-stress behavior of poly(vinyl chloride) and polycarbonate in the glass transition region. J. Polym. Sci..

[57] Chau JLC (1981). Fracture of shear bands in atactic polystyrene. J. Mater. Sci..

[58] Kulawansa DM, Langford SC, Dickinson JT (2011). Scanning tunneling microscope observations of polymer fracture surfaces. J. Mater. Res..

